# Chemical stability study of diuretics in a concomitant simple suspension with magnesium oxide

**DOI:** 10.1186/s40780-026-00579-x

**Published:** 2026-05-02

**Authors:** Ginjiro Kato, Hidemichi Mitome, Mayu Terada, Noriaki Hidaka, Mamoru Tanaka, Kazuki Akira

**Affiliations:** 1 Laboratory of Pharmaceutical Analytical Chemistry, College of Pharmaceutical Sciences, Matsuyama University, 4-2 Bunkyo-cho, Matsuyama, Ehime, 790-8578 Japan; 2 Division of Pharmacy, Ehime University Hospital, 454 Shitsukawa, Toon, Ehime, 791-0295 Japan

**Keywords:** Simple suspension method, Magnesium oxide, Spironolactone, Eplerenone, Diuretics, Stability, Degradation, HPLC, Mass spectrometry.

## Abstract

**Background:**

A simple suspension method has been widely used in Japan, however, chemical interactions among drugs in co-suspensions have not been fully clarified. Magnesium oxide (MgO), which is frequently prescribed as a laxative, can interact with various drugs. Eplerenone and spironolactone, potassium-sparing diuretics, are sometimes co-prescribed with MgO to older patients. In this study, we investigated the chemical stability of eplerenone and spironolactone in a co-suspension with MgO and characterized the structures of the degradation products that were formed under the conditions.

**Methods:**

An eplerenone or spironolactone tablet was soaked with or without an MgO tablet in warm water in a tube according to a standard simple suspension method. The contents in the tube were mixed by inversion after 10 min, 1 h or 5 h to prepare a simple suspension. In separate experiments, the suspension prepared after 10 min soaking was allowed to stand for 50 min or 4.8 h at room temperature. The suspensions were immediately analyzed by high-performance liquid chromatography. The recovery rates of the diuretics from the suspensions were calculated relative to the labeled amounts. The degradation products were isolated and the structures analyzed by high-resolution mass spectrometry.

**Results:**

The high-performance liquid chromatographic analysis showed that the diuretics were stable in their simple suspensions without MgO under any of the conditions. When co-suspended with MgO, a slight degradation was observed for eplerenone after just 10 min soaking and the degradation was statistically significant after 5 h soaking, whereas spironolactone was stable even after 5 h soaking. On the other hand, when the co-suspensions with MgO were left alone after mixing, eplerenone significantly degraded in 50 min, and spironolactone slightly degraded in the same period. Based on the mass spectra from the degradation products, hydrolysis of the lactone ring was shown to have occurred in both diuretics co-suspended with MgO. For spironolactone, hydrolysis and elimination of the thioester were also shown to have occurred in the co-suspensions.

**Conclusions:**

Eplerenone is more unstable than spironolactone in the simple co-suspension with MgO. As such, the simple co-suspensions of eplerenone are preferably prepared immediately before administration.

**Supplementary Information:**

The online version contains supplementary material available at 10.1186/s40780-026-00579-x.

## Background

**Fig. 1:**
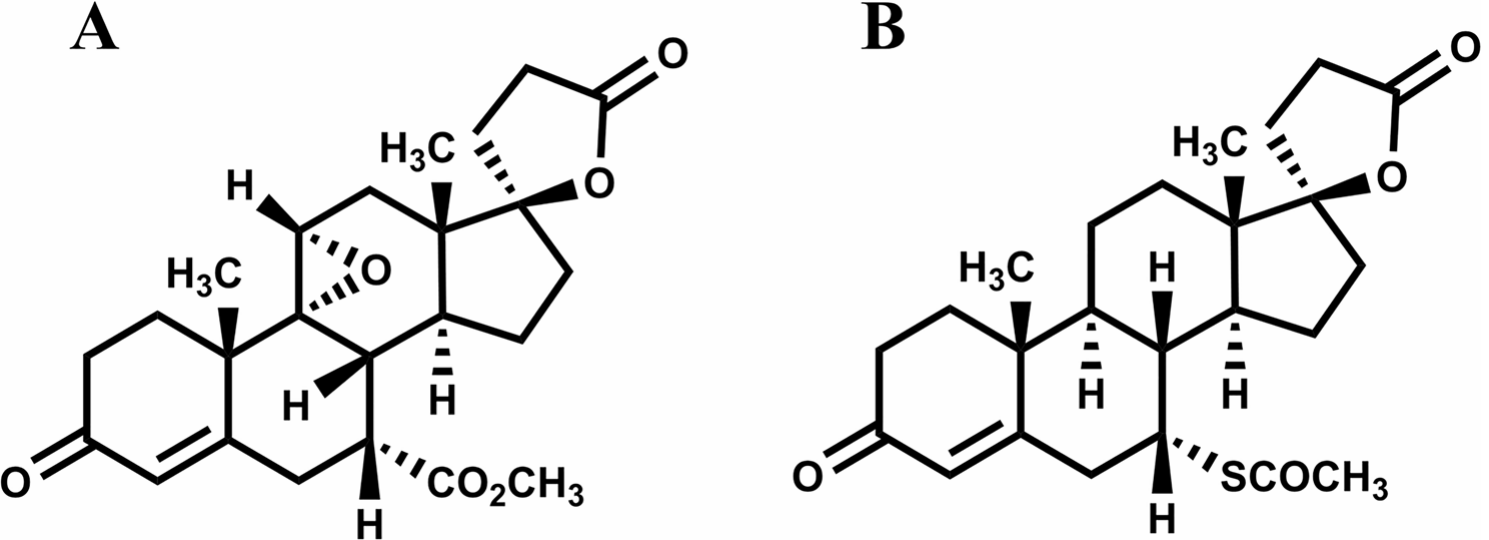
Chemical structures of eplerenone (**A**) and spironolactone (**B**)

A simple suspension method (SSM) has been widely used for patients with dysphagia, particularly older ones in Japanese clinical settings [1]. Although an SSM is superior to a conventional crushing method, a major concern is the chemical stability of drugs simultaneously suspended with acidic or alkaline ones [2]. In East Asia, magnesium oxide (MgO) as a laxative has been generally prescribed to older patients, and thus MgO is sometimes taken together with other drugs [3, 4]. Since MgO is slightly soluble in water to show basicity and interacts with various drugs [5–7], it is important to examine the chemical stability of major drugs in a concomitant simple suspension with MgO (hereafter simply called ‘co-suspension’) [8–10]. Several potassium-sparing diuretics (aldosterone antagonists) are widely used for the treatment of hypertension and heart failure, which are prevalent in the elderly [11, 12]. Thus, these drugs can sometimes be co-prescribed with MgO to older patients. Among the drugs, eplerenone and spironolactone have been more extensively used for many years with a substantial body of clinical experience [13–15]. In addition, these two drugs have alkali-labile functional groups, i.e. lactone and esters (Fig. 1).

In this paper, the chemical stability of eplerenone and spironolactone was investigated in the co-suspension with MgO, and their degradation products were analyzed using high-resolution mass spectrometry.

## Materials and methods

### Materials

Selara Tablets 25 mg (Viatris, PA, USA), Aldactone^®^-A Tablets 25 mg (Pfizer, NY, USA), and Magmitt Tablets 330 mg (Nihon Shinyaku, Kyoto, Japan) were used as the formulations of eplerenone, spironolactone, and MgO, respectively. The formulations, Selara, Aldactone, and Magmitt, are hereafter referred to as EPL, SPRL, and MG, respectively. Distilled water, acetonitrile, methanol, formic acid, and chloroform were purchased from Fujifilm Wako Chemicals (Osaka, Japan). The solvents were of high-performance liquid chromatography (HPLC) grade, except for chloroform.

### Preparation and treatment of simple suspensions

EPL or SPRL (one tablet) was soaked with or without MG (one tablet) in 20 mL of distilled water (55 °C) in a centrifuge tube, according to the standard SSM [1]. The tube was allowed to stand without mixing at room temperature for 10 min, 1 h, or 5 h, and the contents were immediately mixed with inversion 15 times to prepare a suspension. In separate experiments, the suspension mixed after 10 min soaking was allowed to stand for 50 min or 4.8 h before analysis at room temperature. The suspensions were treated for HPLC analysis, in which the diuretics were extracted and neutralized according to a previously described method for amoxicillin [9]. However, acetonitrile (30 mL) was used for the extraction, and the solution after neutralization totaled 100 mL with it. The solution was diluted with acetonitrile by a factor of 20 before injection into the HPLC system. The suspensions were prepared three times under each condition.

The supernatant of the simple suspension prepared after 10 min soaking was subjected to pH measurement using InLab Routine Pro (Mettler Toledo, Ohio, USA). The supernatant was obtained by centrifugation for 5 min, immediately after suspension preparation. The pH meter was calibrated at room temperature using standard buffer solutions (pH 4.01, 6.86, and 9.18) before measurements. The suspension preparation for pH measurement was also performed three times in any of the conditions.

### HPLC analysis of simple suspensions

An HPLC system with a photodiode array detector (Shimadzu, Kyoto, Japan) was used [16]. A reversed-phase Atlantis T3 column (250 × 4.6 mm i.d., 5 μm; Waters, Milford, USA) fitted with a guard column (20 × 4.6 mm i.d., 5 μm) was used. The column oven was set at 40 °C. The injection volume was 10 µL. Other HPLC conditions are summarized in Table 1. The concentrations of the diuretics were measured using an absolute calibration method based on the peak areas. The amounts of diuretics in the simple suspensions were determined from the concentrations, and the percentages of the measured values to the labeled amounts of the formulations (recovery rates) were calculated. The HPLC method was validated for linearity, accuracy, precision, limit of detection, and limit of quantification (Tables S1 and S2).

**Table 1:** HPLC conditions

Formulation	Mobile phase^a^	Wavelength (nm)^b^
EPL	0.1% formic acid/methanol = 50/50	242
SPRL	0.1% formic acid/methanol/acetonitrile = 35/45/20	238 and 288

^a^The flow rate was 1.2 mL/min

^b^Wavelengths used to draw the chromatograms

### Structural analysis of degradation products from eplerenone and spironolactone

The degradation product of eplerenone, **E1**, was extracted with chloroform from the aqueous supernatant of the co-suspension of EPL. The organic layer was evaporated to dryness, and the residue redissolved in acetonitrile. The resulting solution was analyzed by high-resolution electrospray ionization mass spectrometry and mass spectrometry/mass spectrometry (MS/MS) using a micrOTOF-Q mass spectrometer (Bruker Daltonics, Kanagawa, Japan).

The aqueous supernatant of the co-suspension of SPRL was evaporated to dryness and redissolved in acetonitrile. The solution was injected into the above HPLC apparatus equipped with an Atlantis T3 column (250 × 10 mm i.d., 5 μm; Waters, Milford, USA) with a guard column (10 × 10 mm i.d., 5 μm). The flow rate was set to 5.0 mL/min. The fractions of the degradation products, **S1-S5**, were collected, followed by evaporation to dryness. The residues were redissolved with acetonitrile for **S1-S4** and 0.1% formic acid-acetonitrile (1/1) for **S5**, followed by mass spectrometric analysis as described above.

### Statistical analysis

All statistical analyses were performed using one-way ANOVA followed by Tukey’s test to determine significance. The threshold for assessing significance was *p* < 0.05.

## Results

Under the SSM conditions, the diuretic formulations and MG were completely disintegrated, and the suspensions could be prepared under any of the conditions. The pH values of the aqueous supernatants from the simple suspensions without MG were neutral, and those of the co-suspensions were weakly alkaline (Table 2).

**Table 2:** pH values of the supernatants of the simple suspensions prepared after 10 min soaking^a^

Formulation	pH	
	without MG	with MG
EPL	6.8 ± 0.18	10.6 ± 0.02
SPRL	6.9 ± 0.08	10.7 ± 0.01

^a^When only MG (one tablet) was subjected to the SSM, the pH values of the suspensions were 10.7 ± 0.10. The values are shown as mean ± standard deviation. Temperatures of the supernatants during pH measurements were in the range of 25 to 28 °C

**Fig. 2:**
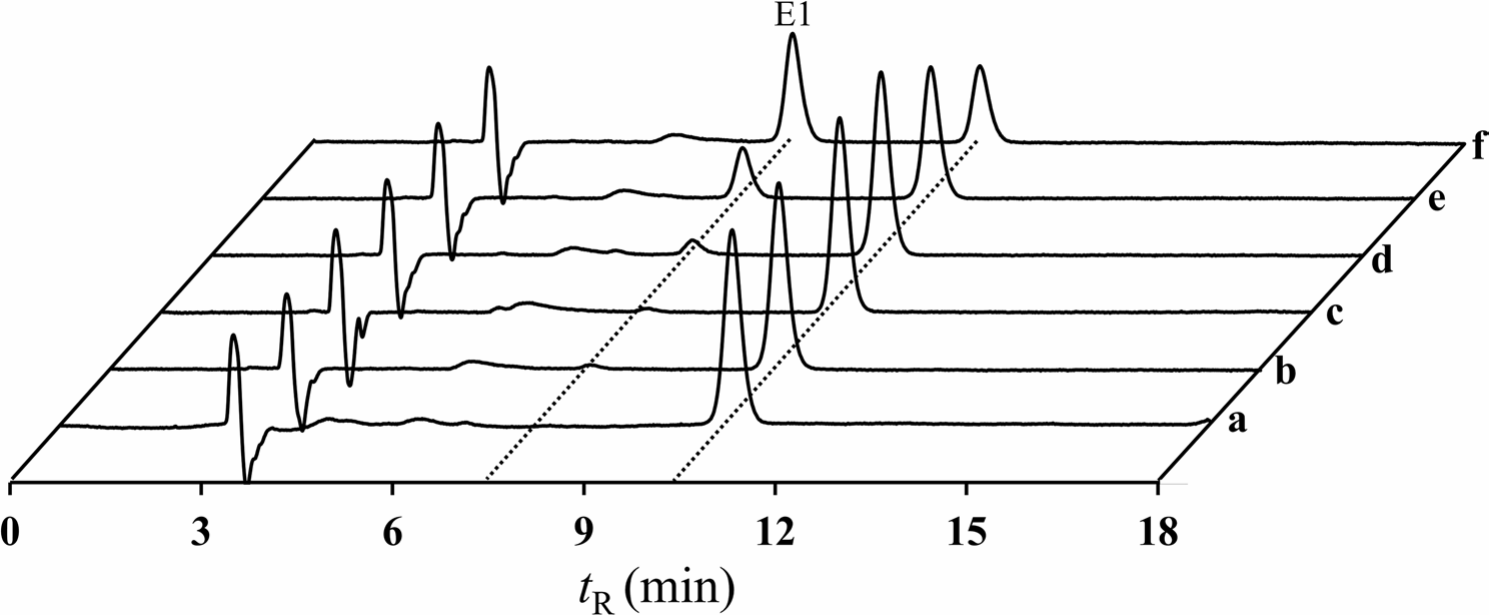
HPLC chromatograms of the simple suspensions of EPL. The peak at *t*_R_ 10.4 min is due to eplerenone. E1 indicates the peak of the degradation product. The conditions for the preparation of the suspension were as follows. **a**: EPL only, 10 min soaking before mixing; **b**: EPL + MG, 10 min soaking before mixing; **c**: EPL + MG, 1 h soaking before mixing; **d**: EPL + MG, 5 h soaking before mixing; **e**: EPL + MG, left for 50 min after mixing; **f**: EPL + MG, left for 4.8 h after mixing

**Fig. 3:**
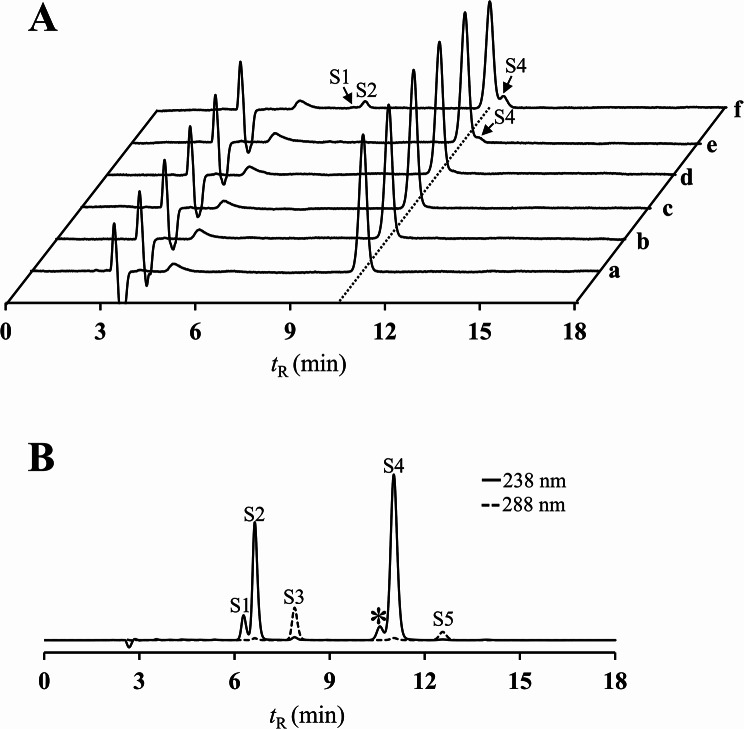
HPLC chromatograms of the simple suspensions of SPRL. The major peak at *t*_R_ 10.6 min is due to spironolactone. S1-S5 indicate the peaks of the degradation products. **Panel A**; The conditions for the preparation of the suspension were as follows. **a**: SPRL only, 10 min soaking before mixing; **b**: SPRL + MG, 10 min soaking before mixing;** c**: SPRL + MG, 1 h soaking before mixing; **d**: SPRL + MG, 5 h soaking before mixing; **e**: SPRL + MG, left for 50 min after mixing;** f**: SPRL + MG, left for 4.8 h after mixing. The chromatograms were drawn at 238 nm. **Panel B**; The co-suspension of SPRL was prepared as in** f** (panel A), and the supernatant was directly analyzed. The chromatograms were drawn at 238 and 288 nm. The peak with an asterisk is due to spironolactone

The HPLC chromatograms obtained from the simple suspensions are shown in Figs. 2 and 3. In the simple suspension of EPL or SPRL alone, only the peak due to eplerenone (*t*_R_ 10.4 min) or spironolactone (*t*_R_ 10.6 min) was observed.

In the co-suspension, the results of the HPLC analysis varied depending on the diuretics and conditions (Figs. 2 and 3). In the co-suspension of EPL, a very small degradation peak (*t*_R_ 7.5 min, **E1**) was observed with a major peak of eplerenone after only 10 min soaking. In contrast, for SPRL, only one peak due to spironolactone was observed, even when the soaking time without mixing was extended to 5 h.

When the co-suspensions of EPL were left alone after mixing, the eplerenone peak significantly decreased in 50 min and 4.8 h, with the increased degradation peak due to **E1** (Fig. 2). On the contrary, for the co-suspension of SPRL, a minor degradation peak (*t*_R_ 11.0 min) was observed in 50 min after mixing, and two additional peaks (*t*_R_ 6.3, 6.6 min) were observed in 4.8 h after mixing (Fig. 3A). Very small degradation peaks (*t*_R_ 7.9, 12.6 min) were also observed at 288 nm. When the aqueous supernatant of the co-suspension left for 4.8 h after mixing was analyzed by HPLC without any pretreatment, the five degradation peaks were clearly detected at 238 or 288 nm (Fig. 3B). The degradation products with retention times at 6.3, 6.6, 7.9, 11.0, and 12.6 min are hereafter referred to as **S1**, **S2**, **S3**, **S4**, and **S5**, respectively.

The recovery rates of eplerenone and spironolactone from the simple suspensions were determined based on the HPLC peak area (Table 3). The values were consistent with the above qualitative HPLC results.

**Table 3:** Quantitative determination of eplerenone and spironolactone in the simple suspensions^a^

Formulation	without MG				with MG		
	10 min	1 h	5 h		10 min	1 h	5 h
EPL	101.4 ± 2.1	101.2 ± 1.9	99.4 ± 2.5		95.9 ± 1.8^*^	97.7 ± 1.4	92.6 ± 0.2^†^
		(98.4 ± 2.3)	(99.1 ± 0.4)			(69.5 ± 1.3)^#^	(38.6 ± 2.5)^£^
SPRL	101.8 ± 1.1	98.4 ± 2.2	100.1 ± 2.1		99.2 ± 1.1	97.0 ± 1.5	98.1 ± 1.7
		(97.3 ± 0.7)	(97.5 ± 0.9)			(96.8 ± 1.3)	(91.1 ± 4.4)^£^

^a^The recovery rates are shown as mean ± standard deviation. The recovery rates in the parentheses were obtained from the suspensions, which were prepared by mixing after 10 min soaking of the drugs and left for 50 min (1 h in total) or 4.8 h (5 h in total)

^*^p < 0.05 vs. 10 min soaking without MG

^†^*p* < 0.05 vs. 5 h soaking without MG

^#^*p* < 0.05 vs. 50 min after mixing without MG

^£^*p* < 0.05 vs. 4.8 h after mixing without MG

To elucidate the chemical structures of the degradation products in the co-suspensions, their precise molecular weights were measured and the molecular formulas determined using the high-resolution mass spectra (Fig. S1). The structural formulas were deduced based on the differences of the molecular weights between the degradation products and the parent compounds (Table 4). The product ion spectra could be obtained by MS/MS for **E1** and **S1**-**S3**, which confirmed the existence of the functional groups in the respective estimated structures (Fig. S1).

**Table 4:** Chemical structures of the degradation products based on accurate mass measurements

Symbol	Name	Structure	Formula	Molecular weight	m/z		
					Experimental mass	Calculated mass	Error (mDa)
E1	eplerenone acid		C_24_H_32_O_7_	432	431.2070	431.2075	0.6
S1	(7α)-7-(Acetylthio)-17-hydroxy- 3-oxopregn-4-ene-21-carboxylic acid		C_24_H_34_O_5_S	434	433.2046	433.2054	0.9
S2	7α-mercapto-17-hydroxy-3-oxo- 17α-pregn-4-ene-21-carboxylic acid		C_22_H_32_O_4_S	392	391.1925	391.1949	2.3
S3	canrenoic acid		C_22_H_30_O_4_	358	357.2067	357.2071	0.4
S4	7α-thio-spironolactone		C_22_H_30_O_3_S	374	373.1838	373.1843	0.5
S5	canrenone		C_22_H_28_O_3_	340	341.2123	341.2111	-1.2

Accurate mass spectra of **E1** and **S1–S4** were obtained in negative ion mode, whereas that of **S5** was obtained in positive ion mode

## Discussion

Simple suspensions are basically prepared with 10 min soaking immediately before administration. However, the soaking time before mixing is sometimes extended depending on the work situation in clinical settings. Thus, we also prepared the simple suspensions after soaking for 1 or 5 h which was presumed to be the maximum time. As a result of the HPLC analysis of the co-suspensions, eplerenone was found to be somewhat unstable even in the standard soaking time, whereas spironolactone was stable even after a much longer soaking time. In addition, in the co-suspensions after mixing, the decline in stability was recognized for both diuretics. The reason is that the disintegration of tablets is completed by mixing and collisions between particles are increased. Eplerenone was more unstable than spironolactone probably because of its higher water solubility (eplerenone: 9 mg/L, spironolactone: 2 mg/L) [17, 18].

As a result of the high-resolution mass spectrometric analysis of **E1**, there was a possibility that eplerenone degraded by hydrolysis of the lactone moiety or cleavage of the epoxide moiety because the molecular weight of **E1** was 18 (water) more than that of eplerenone. On the other hand, the MS/MS spectrum of** E1** showed a fragment ion probably due to decarboxylation. In addition, Du et al. [19] have reported that the main degradation product of eplerenone in a strong alkaline aqueous solution was a lactone-ring opened form, eplerenone acid. Thus, **E1** was considered to be eplerenone acid. Eplerenone acid **E1** is pharmacologically inactive due to the absence of the lactone moiety [20]. However, **E1** may be converted back to eplerenone after administration, owing to changes in environmental pH values in the body and/or possible enzymatic reactions [21]. On the other hand, the gastrointestinal absorption of **E1** may be reduced when compared with eplerenone since it is a less lipophilic hydroxy acid. In any case, attention should be paid to the chemical stability of eplerenone in the co-suspensions.

Although spironolactone has been reported to be unstable in alcoholic aqueous solutions with strong alkalinity [22, 23], the structures of the degradation products have remained unknown. As a result of the mass spectrometric analysis, hydrolysis (**S2**, **S4**) and elimination (**S3**, **S5**) of the thioester were shown to have occurred in addition to hydrolysis (**S1**-**S3**) of the lactone moiety. To the best of our knowledge, the chemical structures for **S1** and **S2** have not been described in the literature. The compounds **S3-S5** are known to be metabolites of spironolactone in humans [24]. **S4** and **S5** have been reported as impurities in the drug formulation [25]. **S3** does not exhibit aldosterone antagonistic activity in vitro, however, it is converted to pharmacologically active **S5** in vivo. The activity of **S5** is comparable to spironolactone, whereas that of **S4** is weaker than spironolactone [26].

## Conclusions

In the co-suspension, eplerenone is relatively unstable, and the co-suspensions are thus desirable to be prepared immediately before administration. In contrast, spironolactone is more stable than eplerenone under the same conditions, and thus suitable for the co-suspension. It should be noted that the diuretics are more unstable in the co-suspensions after mixing.

## Supplementary Information

Below is the link to the electronic supplementary material.

Supplementary Material 1

## Data Availability

The datasets used and/or analyzed during the current study are available from the corresponding author on reasonable request.

## Abbreviations

EPL: Selara tablets 25 mg
HPLC: high-performance liquid chromatography
MG: Magmitt tablets 330 mg
MS/MS: mass spectrometry/mass spectrometry
SPRL: Aldactone ®-A tablets 25 mg
SSM: simple suspension method
