# Supplementary material for: Chemical stability study of diuretics in a concomitant simple suspension with magnesium oxide
Source: J Pharm Health Care Sci. 2026 May 2;12:58. doi: 10.1186/s40780-026-00579-x (PMC13285385; doi:10.1186/s40780-026-00579-x)
Supplement: Supplementary file 1 — Supplementary Material 1 [file 40780_2026_579_MOESM1_ESM.docx]

**Table S1 Calibration curve, LOD, and LOQ^a^**

| Drug | Slope | Intercept | *r* ^2^ | Concentration range (μg/mL) | LOD (ng/mL) | LOQ  (ng/mL) |
| --- | --- | --- | --- | --- | --- | --- |
| spironolactone | 22613 | -977 | 0.9997 | 0.625-10.0 | 25.4 | 77.1 |
| eplerenone | 19108 | 300 | 0.9999 |  | 77.0 | 233 |

^a^LOD and LOQ mean limit of detection and limit of quantification, respectively.

**Table S2 Intra- and inter-day variations^a^**

| Drug | Concentration (μg/mL) |  | Intra-day (*n*=3) | | |  | Inter-day (*n*=3) | | |  |
| --- | --- | --- | --- | --- | --- | --- | --- | --- | --- | --- |
|  |  |  | Mean (μg/mL) | R.S.D.^b^ (%) | Bias^c^ (%) |  | Mean (μg/mL) | R.S.D.^b^ (%) | Bias^c^ (%) | |
| spironolactone | 0.625 |  | 0.648 | 2.1 | 3.7 |  | 0.642 | 1.1 | 2.7 | |
|  | 5.0 |  | 4.9 | 1.1 | -1.4 |  | 5.0 | 0.4 | -1.0 | |
|  | 10.0 |  | 10.0 | 0.3 | 0.3 |  | 10.1 | 0.4 | 0.8 | |
| eplerenone | 0.625 |  | 0.596 | 0.6 | -4.6 |  | 0.600 | 0.6 | -3.9 | |
|  | 5.0 |  | 4.9 | 0.5 | -1.4 |  | 5.0 | 1.3 | -0.1 | |
|  | 10.0 |  | 10.1 | 0.4 | 0.9 |  | 10.0 | 1.1 | 0.01 | |

^a^The quality control samples were analyzed five times using the HPLC system for the intra-day precision, and such experiments were performed in three different days for the inter-day precision.

^b^R.S.D means relative standard deviation.

^c^The differences between the sample concentrations and the measured ones.


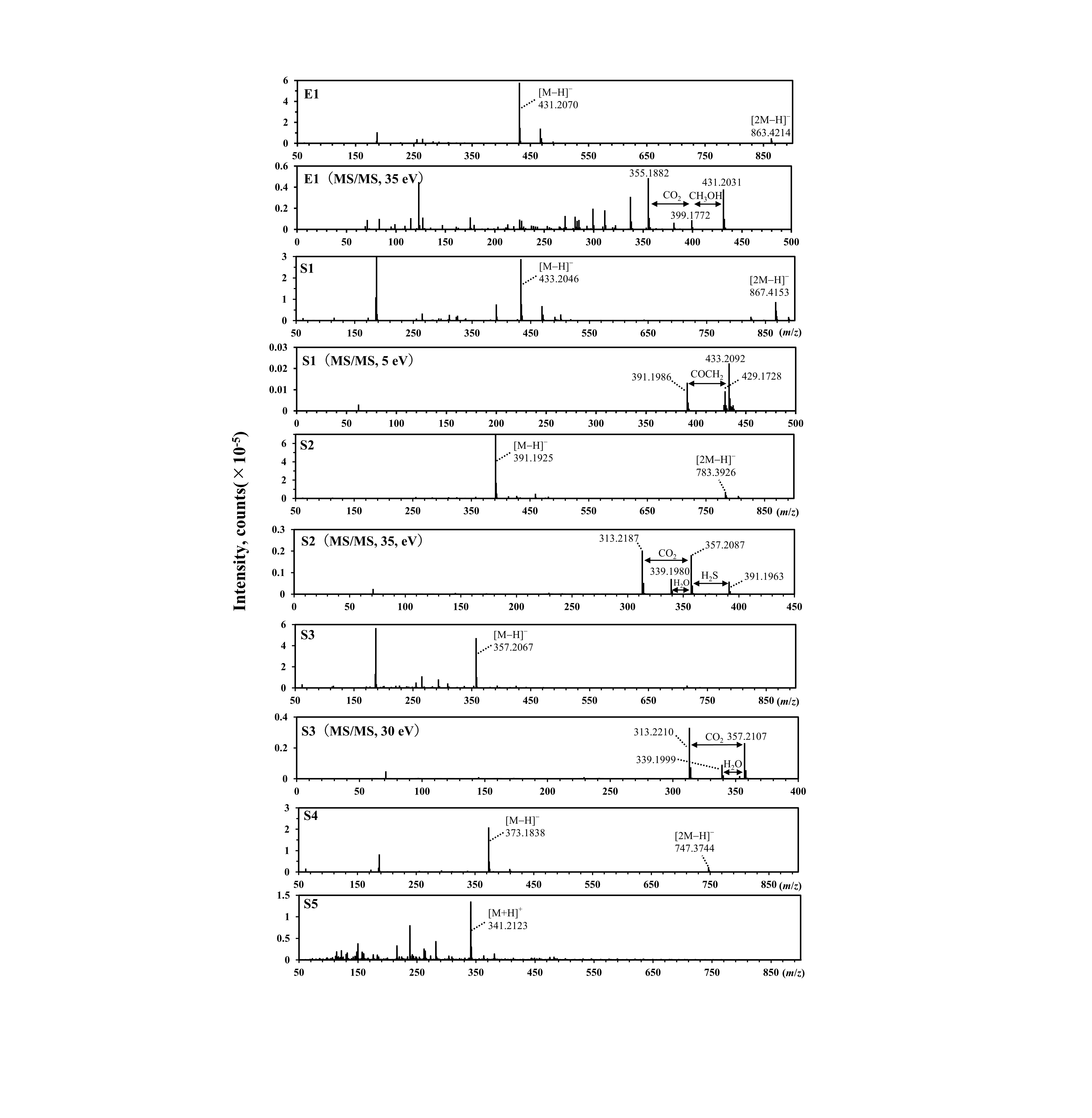


**Fig. S1　Accurate mass spectra and MS/MS spectra of the degradation products.**

Accurate mass spectra and MS/MS spectra (product ion spectra) of **E1** and **S1–S4** were measured in negative ion mode, whereas those of **S5** were measured in positive ion mode. No fragment ions were observed in the MS/MS spectra of **S4** and **S5**. The collision energy is indicated in the parenthesis of each spectrum.
